# College and Hospital Notes

**Published:** 1903-02

**Authors:** 


### COLLEGE AND HOSPITAL NOTES.

The Alumni Association of the medical department, University of Buffalo, will hold its mid-winter session at the University building, Friday evening, February 23, 1903. This promises to be one of the most enjoyable and instructive occasions which have been held since the winter meetings were instituted. The new quarters of the physiological department will be formally opened at that time, and the speaker of the evening will be one of the foremost physiologists in this country. A new story has been added to the west wing of the college building for the accommodation of the physiological and pharmacological departments, with a new and complete equipment for laboratory instruction and research in these fields of work. The opportunities for teaching in these topics is thus placed on a par with other great schools in this country. The name of the speaker of the evening will be announced as soon as arrangements are completed.

---

The Buffalo Hospital of the Sisters of Charity, at the annual meeting held January 14, elected the following-named officers of the hospital staff, to serve for the ensuing year: Edward J. Meyer, president; Charles S. Jewett, vice-president; Alfred H. Diehl, secretary and treasurer.

Immediately after election the officers were installed. Following the installation the members adjourned to one of the wards on the second floor of the hospital building, where a banquet was held.

---

The Emergency Hospital staff has been enlarged by the appointment of the following-named physicians: E. J. Meyer and E. M. Dooley, in the surgical service; L. G. Hanley, in the gynecological service; J. J. Mooney, laryngological service; M. Keiser, ear diseases; B. H. Daggett, genitourinary service. Otherwise the staff remains as before.

---

The Lexington Heights Hospital was opened in 1890, being one of the earlier private hospitals in this city. It was then known as the Wilcox Private Hospital, with Dr. DeWitt G. Wilcox as proprietor and owner, who organised it for the purpose of providing a place for his surgical patients. It was organised as a stock company in 1893. Later it was enlarged and called the Lexington Heights Hospital. It was conducted as a stock company until 1897, when Dr. Wilcox leased the property from the company and used it for his own patients. Dr. Wilcox has now purchased the entire plant, buildings and lot, with all its equipment, and is again the sole owner and proprietor. It will still be known as the Lexington Heights Hospital. It has been newly furnished throughout and equipped with practical appliances for the treatment of gynecological and surgical diseases, and a complete *x*-ray outfit has been added. A regularly incorporated training school is attached to the hospital, which has graduated nurses for the past ten years.

---

The Washington Post-Graduate Medical School began its first term January 12, 1903, under the presidency of General George M. Sternberg, M. D., LL.D., Surgeon-General U. S. Army, retired. A full corp of teachers has been organised, and instruction will be given on preventive medicine, comparative medicine, animal parasites, serum-therapy, biochemistry, sanitary chemistry, bacteriology, pathology, internal medicine, applied anatomy, surgery, Röntgen-ray work, military and naval medicine and surgery, orthopedic surgery, gynecology, obstetrics, mental and nervous diseases, tropical diseases, diseases of children, diseases of the stomach, diseases of the eye, of the nose, throat and ear, venereal and genitourinary diseases, and diseases of the skin. The hospitals of Washington will be open for clinical teaching to matriculants, and the attractions of the National capital are such as to justify expectations of a large attendance.

---

The Olean General Hospital was opened November 12, 1902, with accommodations for 16 patients. It is established on what was known as the Dean property, which was purchased less than a year ago for $6,000. The refitting cost between $3,000 and $4,000, which has made it a modern institution, equipped with preparatory and operating rooms, wards, and rooms for private patients. A number of the citizens of Olean contributed a fund of $6,000 to guarantee the salary of the matron for 10 years and the hospital opened free of debt. The physicians in active practice, about fifteen in number, fitted up the operating rooms, and the wards and other rooms were furnished by different church and fraternal societies; the employees of the Pennsylvania Railroad Company also contributed to that end. The citizens, and especially the physicians, of Olean, are to be congratulated upon the establishment of the hospital on a sound basis.

---

THE New York School of Clinical Medicine has established a special department of neurology, of which Dr. T. D. Crothers, of Hartford, Conn., has been elected professor, for the study of the neuroses and psychoses of alcoholism and of drug habits. Dr. Crothers is announced to deliver immediately a course of clinical lectures on inebriety from alcohol, opium, chloral, cocaine and other narcotics. These lectures appear to be timely, for the diseases dependent upon or associated with the abuse of alcohol, opium, chloral, cocaine, and other narcotic drugs are steadily increasing in the United States, and the demand for special treatment in institutions and retreats is becoming more pressing every year.

